# Omics Technologies Improving Breast Cancer Research and Diagnostics

**DOI:** 10.3390/ijms241612690

**Published:** 2023-08-11

**Authors:** Arianna Orsini, Chiara Diquigiovanni, Elena Bonora

**Affiliations:** Department of Medical and Surgical Sciences (DIMEC), University of Bologna, 40131 Bologna, Italy; arianna.orsini8@unibo.it (A.O.); elena.bonora6@unibo.it (E.B.)

**Keywords:** breast cancer, omics technologies, tumor heterogeneity, biomarkers

## Abstract

Breast cancer (BC) has yielded approximately 2.26 million new cases and has caused nearly 685,000 deaths worldwide in the last two years, making it the most common diagnosed cancer type in the world. BC is an intricate ecosystem formed by both the tumor microenvironment and malignant cells, and its heterogeneity impacts the response to treatment. Biomedical research has entered the era of massive omics data thanks to the high-throughput sequencing revolution, quick progress and widespread adoption. These technologies—liquid biopsy, transcriptomics, epigenomics, proteomics, metabolomics, pharmaco-omics and artificial intelligence imaging—could help researchers and clinicians to better understand the formation and evolution of BC. This review focuses on the findings of recent multi-omics-based research that has been applied to BC research, with an introduction to every omics technique and their applications for the different BC phenotypes, biomarkers, target therapies, diagnosis, treatment and prognosis, to provide a comprehensive overview of the possibilities of BC research.

## 1. Introduction

The most common malignant tumor in the world and the leading cause of mortality for women is breast cancer (BC), which accounts for 6.6% of all cancer deaths worldwide [1]. With an expected 2.3 million new cases worldwide, female BC has surpassed lung cancer as the most diagnosed malignancy, according to GLOBOCAN 2020 [1].

Currently, early detection and accurate diagnosis are the keys to effective BC management [2]. BC is frequently found via core needle biopsy or fine-needle aspiration biopsy, and, in uncertain cases, tissue biopsy is performed to confirm specific pathological characteristics [3]. Nevertheless, metastases or resistance to chemotherapy can develop in up to 30% of women who are initially diagnosed with cancer at the early stages. The conventional course of treatment for locally advanced BC is either surgery or mastectomy, with or without radiation. Although immunotherapy is one of the most promising cancer therapy choices, only a small subset of responding individuals have shown long-term therapeutic benefits [4]. 

**Figure 1:**
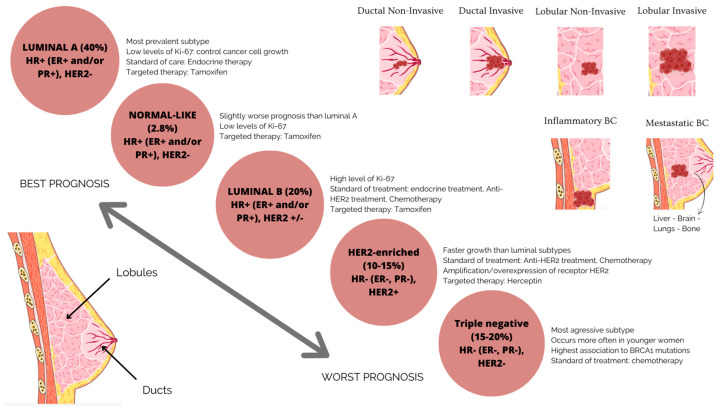
BC subgroups are based on molecular and histological characteristics. Luminal BC is an ER-positive, PR-positive, HER2-negative tumor type with low levels of the proliferation marker Ki67. The tumor has a good prognosis and a modest rate of growth. The tumor luminal BC has high levels of Ki67, which accelerates its growth. It is ER-positive, PR-positive and HER2-negative or -positive. This suggests a less favorable prognosis than forms indicated in luminal A. HER2-enriched or positive carcinoma has a poor prognosis, is more aggressive than luminal BC and is ER-negative, PR-negative and HER2-positive. Drugs that specifically target the HER2 protein can successfully treat it. One of the more aggressive subtypes of BC is triple-negative or basal BC, which lacks the expression of the ER, PR and HER2 proteins.

Numerous studies have demonstrated the importance of defining BC’s molecular subtypes, well known for its heterogeneity in molecular properties and cellular makeup, in the diagnosis, treatment and prognosis of the disease [5,6,7], as well as in different therapeutic responses [8]. Shorter overall survival and disease-free survival are related to tumor heterogeneity, with the expression of hormone receptors, such as the human epidermal receptor 2 (HER2+), progesterone receptors (PR+) and estrogen receptors (ER+), determining the molecular subtypes of BC (Figure 1) [9,10]. 

## 2. Methods

A comprehensive literature review was conducted using the PubMed (https://pubmed.ncbi.nlm.nih.gov/ (accessed on 8 August 2023)) and Medline (https://www.nlm.nih.gov/ (accessed on 8 August 2023)) databases to identify studies discussing omics research for BC evolution and progression. Titles and abstracts of studies published in English between 2010 and 2023 were reviewed and relevant articles obtained in full versions. Herein, we explore different techniques and their applications to investigate the different BC phenotypes, biomarkers, target therapies, diagnosis, treatment and prognosis. 

## 3. Omics Approaches to Uncover BC Alterations

**Figure 2:**
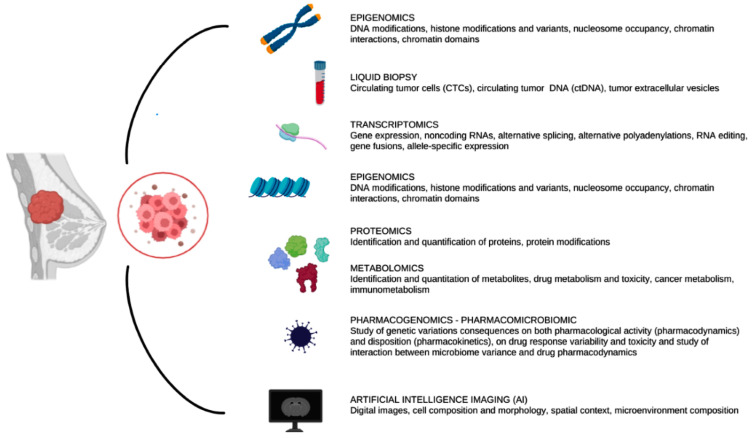
Overview of the multi-omics technologies that we describe in the following sections. These technologies could be invasive or non-invasive for patients and the analysis could start from surgical specimens or imaging of the tumor.

Cancer etiology depends on complex interplays at the genomic, transcriptional, proteomic and metabolic levels [11]. An in-depth investigation of the tumor at the omics level is required, for a thorough characterization of the cancer biology, plasticity and heterogeneity. Integrative multi-omics analysis has significantly improved our understanding of the complex nature of tumor biology, including tumor evolution, tumor heterogeneity, the tumor microenvironment, immune evasion and drug resistance [12]. The potential benefit of the omics technologies in cancer research is vast, since they offer an unmatched opportunity to define cancer biology at many pathological and molecular levels [13]. Indeed, combining multi-omics data is a crucial first step to uncover the underlying workings of oncogenesis. Numerous omics approaches, such as genomics, epigenomics, transcriptomics, proteomics and metabolomics, are available to analyze various yet complementing biological layers with high-throughput technological advances (Figure 2) [14]. 

Here, we summarize the omics applied in BC research and the emerging results.

### 3.1. Liquid Biopsies, Transcriptomics and Epigenomics

#### 3.1.1. Liquid Biopsy 

A liquid biopsy (LB) is a simple and non-invasive method that allows medical professionals to learn a large amount of information about a tumor from a small blood sample. Liquid biopsies involve collecting tumor-derived entities from the body fluids of cancer patients, such as circulating tumor cells (CTCs), circulating tumor DNA (ctDNA), tumor extracellular vesicles, etc., and analyzing the genomic and proteomic information that they carry. The analysis of these components allows us to monitor cancer progression in real time. LB is already a well-established technique in metastatic BC treatment, since clinicians use it to characterize the disease, for decision making and the improvement of patient outcomes.

We report herein several examples of how liquid biopsies are applied to BC studies.

- Early detection and patient stratification

LB can help to detect BC at an early stage by searching for circulating biomarkers or indicators in a laboratory test.

In cases of early-stage BC (EBC), where CTC-positive patients are rare, a thorough analysis of tumor-related analytes in the LB may also be beneficial to determine the disease status [15,16]. The transition from latent to aggressive minimal residual disease (MRD) can be determined by the further molecular characterization of CTCs, with a focus on the most used blood devices that can select and identify CTC enrichment [17,18].

Setayesh et al. used a third-generation high-definition single-cell assay (HDSCA3.0) for LB (LBx) from late-stage BC, EBC and peripheral blood samples from normal donors to simultaneously investigate epithelial, mesenchymal, endothelial and hematopoietic cells and large extracellular vesicles [19]. According to their research, when compared to early-stage patients and normal donors, late-stage patients had a larger concentration of CTCs. Furthermore, they found that early-stage groups had more tumor-associated large extracellular vesicles than late-stage and normal donor groups, proposing the robust identification of uncommon circulating events in peripheral blood draws [19]. 

Cohen et al. combined the detection of somatic mutations found in ctDNA with the analysis of protein biomarkers in plasma to accurately localize the primary site of the cancer; however, this test has low sensitivity [20]. ctDNA testing in high-risk individuals enables tumor subtype prediction and early detection and offers transcriptional information [21]. Moreover, tumor cells of different origins have been shown to harbor specific methylation profiles, enabling the analysis of ctDNA to reveal the BC position [22]. 

The loss of heterozygosity at four polymorphic markers in cfDNA (D13S159, D13S280, D13S282 at region 13q31-33, and D10S1765 at PTEN region 10q23.31) analyzed by PCR-based fluorescence microsatellite analyses also correlated significantly with lymph node status [23].

- LB in Prognosis

To enhance patient outcomes, LB can assist in prognosis definition. For instance, LB can help to predict BC recurrence. Indeed, it was shown that patients with five or more CTCs per 7.5 mL of blood at baseline had shorter median progression-free survival (PFS) and overall survival (OS) compared to those with fewer than five CTCs [24]. Similar results were observed in other studies, showing that patients with five or more CTCs had shorter OS and recurred earlier [16,25].

Numerous studies have investigated how well BC cfDNA concentrations can predict outcomes. For example, it was observed that cfDNA levels rose in patients with malignant lesions and correlated with the tumor size and clinical stage of lymph node metastasis [26,27,28]. 

In daily diagnostic routines, assays to identify *PIK3CA* mutations in patients with HER2-negative, *PIK3CA*-mutated, advanced or metastatic BC and patient-specific multiplexed cancer alteration analysis methods for targeted digital sequencing (TARDIS) for the identification of patients at risk of recurrence are well-known applications of LB [29,30].

- Monitoring

Real-time cancer progression monitoring is possible using LB to search for CTCs. Previous research has established a relationship between the CTC burden in late-stage BC and progression-free survival; however, therapy delivery has proven to have an impact on CTC abundance [25,31]. The serial monitoring of ctDNA levels after treatment has been used to assess and monitor treatment responses, showing a correlation with tumor size changes and survival outcomes [32,33].

- Personalized treatment

Treatment for BC patients can be tailored with the aid of LB. For patients with metastatic BC, LB can assist clinicians in making treatment choices. Multiple studies have focused on the usefulness of LB for BC identification in the context of metastatic BC (MBC), with the goal of improving clinical decision making and enhancing patient outcomes [34,35]. Dynamic changes in CTC levels during treatment have been associated with clinical outcomes, with patients who show a decrease in CTC count experiencing better OS and PFS [36].

LB has been used also for the mutation monitoring of specific genes. In particular, mutations in the *ESR1* gene, which encodes for estrogen receptor a (ER-a), were found in BC patients who received endocrine therapy. Metastatic BC has higher rates of *ESR1* mutation compared to primary BC, and the mutations may be acquired under the pressure of endocrine therapy [37]. The prevalence of *ESR1* variants is higher in patients treated with aromatase inhibitor therapy (AI therapy) for metastatic disease compared to the adjuvant setting [38]. Tay and Tan observed that the profiling of ctDNA for these mutations was more accurate than sequencing the primary tumor and could predict resistance to AI-guided therapy [39]. The serial monitoring of ctDNA for *ESR1* mutations can guide therapy and improve progression-free survival, since, by analyzing ctDNA, variants in *ESR1* can be detected before clinical progression, allowing for early therapeutic intervention.

Despite the initial optimism and expectations following the discovery of CTCs and ctDNA from liquid biopsies in cancer patients, recent data show that although these markers offer a high grade of cancer specificity, these indicators are uncommon in body fluids; for instance, ctDNA accounts for less than 1% of the total circular free DNA (cfDNA) detected in body fluids [40,41]. Moreover, in a meta-analysis of 69 studies with a total of 5736 patients, Lee et al. reported that the ctDNA mutation rates of *TP53*, *PIK3CA* and *ESR1* were roughly 38%, 27% and 32%, respectively, concluding that these rates were too low for application in BC screening [41]. 

#### 3.1.2. Transcriptomics

Transcriptomics analysis consists of sequencing all the coding and noncoding RNAs transcribed in a tissue/cell in physiological and pathological conditions. 

Single-cell DNA/RNA sequencing (scDNA/RNA-seq) is a powerful tool that can provide insights into the cellular and molecular landscape of BC at a single-cell resolution, through the separation of single cells, examination of their genomes or transcriptomes and creation of unique sequencing libraries. In recent years, transcriptomics has provided insights into the molecular mechanisms underlying BC development and progression and the investigation of biomarkers and potential therapeutic targets [42]. Below, we discuss several examples.

- Identification of differentially expressed genes (DEGs)

The broad utilization of RNA sequencing (RNA-seq) technologies has dramatically expanded our knowledge of BC, allowing the identification of DEGs and key signature genes during tumor progression [43,44].

Employing RNA-seq, genes that are expressed at extremely high and low levels can be identified [45,46]. For example, dysregulated genes such as *TP53*, *GAPDH*, *cyclin D1*, *HRAS*, *CDK1, CDC6* and *PCNA*, and the activation of *ERBB2*, *FOXM1*, *ESR1* and *IGFBP2* networks, have been reported in BC tissue compared to control tissue [47]. Moreover, scRNA-seq studies on TNBC identified indicative combinations of the expression of ER, PR, GATA3, E-cadherin and multiple cytokeratins for HER2+ BC, or high levels of Ki-67, p53, EGFR and the hypoxia marker CAIX [48]. 

sc-seq gives also an overview of the different subpopulations in BC, as has been reported by Tokura et al. with the profiling of ductal carcinoma in situ (DCIS) and invasive ductal carcinoma (IDC). The authors identified clusters of gene expression profiles for cell-type-specific markers, segregating the cells into groups such as luminal epithelial cells, proliferating luminal epithelial cells, basal cells, proliferating basal cells, T cells, proliferating immune cells, B cells, plasma cells, macrophages, monocytes, erythrocytes and stromal cells [49]. The differential expression of the extracellular matrix receptor (ECM receptor) was also reported in invasive BC [50].

- Identification of biomarkers and therapeutic targets

Transcriptomics analysis allows us to identify novel molecules that could serve as biomarkers for BC. One example is polyadenylation (APA)—a post-transcriptional change in the 3′UTR that influences tumor cell proliferation by altering the length of the 3′UTR—which can be used as a predictive biomarker of early BC [51,52]. The majority of BC patients have a shortened 3′UTR, which is closely associated with cell proliferation, according to Kim et al. [53]. Another example is the *ETV6* gene, which was found selectively activated in five molecular cellular subtypes as opposed to normal epithelial cells in a study of the sc-seq of 585 malignant cells [54]. Its overexpression is linked to a worse prognosis in TNBC patients, and this raises the possibility that it could serve as a target for chemotherapy.

Changes at the single-cell level in expression profiles could be used as potential therapeutic targets, as with the ones identified in BC stem cells (BCSCs) [55,56]. Alterations in these cells were found to be associated with an increased risk of relapse, so the authors suggested 74 BCSC marker genes as novel targets and prognostic indicators for BC treatment. Another related technology, spatially annotated single-cell sequencing, could provide insights into the spatial organization of cells within breast tumors and help to identify potential therapeutic targets thanks the use of live-cell imaging at a single-cell resolution [57].

- Characterization of intratumor heterogeneity

sc-seq unmasks the heterogeneity of BC cells, including the identification of rare cell populations, the characterization of cell subtypes and the identification of driver mutations [58,59]. Previous sc analyses led to the characterization of the cellular architecture of BC tissue, defining its cellular composition and tissue organization. Another study used single-nucleus RNA sequencing (snRNA-seq) and microarray-based ST to map subclusters of malignant cells and stromal cell types in distinct BC tissue regions [60].

Wang at al. applied a novel method, genome-driven transcriptome (DGTEC), to the TCGA-BC cohort and identified different BC subtypes. This multi-omics approach led the authors to identify a hybrid BC subtype that they called the Mix_Sub subtype, characterized by a poor prognosis—in particular, with low levels of immune cell infiltration and a dysfunctional T-cell response [61].

BC is characterized by alterations in the ECM that can drive subclonal communication, immunological crosstalk and metabolic crosstalk [62,63,64,65]. To disentangle these complex interactions, spatial transcriptomics (ST) has been applied with the aim of visualizing RNA molecule profiles in specific tissue regions [66,67]. The use of ST technology is critical in the study of solid tumors, which develop in a complex environment where communications between tumor cells, immune cells, fibroblasts and the vasculature can affect disease progression [68,69]. Recently, ST in BC led to the identification of different signatures, such as extensive regions of fibrosis or T-cell enrichment in a dense ring of lymphoid cells with strongly expressed macrophage signatures, or a type I interferon response overlapping with regions of T-cell and macrophage subset colocalization [66,70].

scRNA-seq has been applied to investigate various BC subpopulations but also BC adipose cell subpopulations, identifying de-differentiation from adipocytes into multiple cell types, including myofibroblast- and macrophage-like cells [71,72]. To understand the complexity of the tumor immune microenvironment (TIME), specific studies have been conducted on tissue-resident memory T and B cells [73,74], allowing a better understanding of the general characteristics of immune cells in BC, revealing the vast diversity in the immune cells of both the adaptive and innate immune systems [75,76] and tumor-associated fibroblasts (CAFs) [77], or to depict the comprehensive tumor environment by sequencing all the cells isolated from breast tumors [78]. The use of this technique also confirmed the expression of typical marker genes, such as *ESR1*, PGR and ERBB2 for luminal cells, *MKI67* for proliferating cells and *PTPRC* and *CD53* for immune and myeloid cells [49]. This can aid in the development of personalized treatment strategies for breast cancer patients.

- Tools for early diagnosis and pathway analysis

Several tests have been developed during the past ten years based on transcriptome signatures for early BC diagnosis. The Breast Cancer Index evaluated the expression of the genomic grade index genes *BUB1B*, *NEK2*, *CENPA*, *RRM2* and *RACGAP1*, as well as the ratio of the *HOXB13* and *IL17BR* genes [79]. The 70-gene MammaPrint test employs microarray technology to quantify the expression of genes involved in cell-cycle imbalance (15 genes), angiogenesis (12 genes), proliferation and cancer development (11 genes), spread and invasion (8 genes), growth factor signal transduction (6 genes) and susceptibility to apoptosis (2 genes) [80]. Similar tools are the Oncotype DX, which measures genes for proliferation (5), invasion (2), estrogen (4), *HER2* (2), *GSTM1*, *BAG1* and *CD68*, as well as the Prosigna test and genomic grade index [81].

The application of transcriptomics technologies indicates the future development of novel biomarkers and therapeutic interventions targeting common signaling pathways.

- Understanding of treatment response and resistance

In BC patients, transcriptomics analysis can identify gene expression alterations linked to the response or resistance to a treatment. By examining the molecular profiles of patients that underwent neoadjuvant chemotherapy, a recent study identified DEGs that may be utilized as biomarkers for the chemotherapy response and OS [82]. In this study, Barron-Gallardo et al. performed RNA-seq analysis in BC and identified a total of 1985 DEGs [82]. Among these genes, in neoadjuvant chemotherapy resistance patients, the underexpression of *C1QTNF3*, *CTF1*, *OLFML3*, *PLA2R1*, *PODN*, *KRT15* and *HLA-A* and the overexpression of *TUBB* and *TCP1* correlated with lower OS [82].

Kim et al. discovered drug-resistant cancer cells in a scDNA/RNA-seq analysis of eight BC patients, revealing their adaptation to neo-adjuvant chemotherapy [83].

A particular interest has been shown in metastatic TNBC cells, where *B2M*, *CD52*, *PTMA* and *GZMK* are among the most significant DEGs, and functional enrichment showed their potential sensitivity for immunotherapy since the immune-related items were highly enriched in metastatic TNBC cells [84]. Moreover, β2-microglobulin, encoded by *B2M*, is an essential component of MHC class I, and its loss was an indicator of a poor prognosis, including lymph node metastasis, recurrence and therapy resistance [85,86].

Full transcriptome profiling not only identifies DEGs but also all the RNA profile and tumor cell spectrum. Sinicropi et al. established and improved the RNA-seq library chemistry for formalin-fixed paraffin-embedded (FFPE) tissues, as well as bioinformatics and biostatistical approaches. In a cohort of 136 individuals, the study found more than 2000 RNAs strongly related to BC recurrence, by using the RefSeq RNA network enriched in RNAs with the Reactome database (https://reactome.org/ (accessed on 8 August 2023)) [87]. Interestingly, Baldominos et al. recently used single-cell RNA sequencing with a remarkable spatial resolution, unveiling clusters of quiescent cells (QCCs), already known for their resistance to immunotherapy [88]. They profiled infiltrating cells inside and outside the QCC niche, finding genes associated with hypoxia-induced pathways, and discovered worn-out T cells, tumor-protective fibroblasts and defective dendritic cells in BC [88].

Overall, transcriptomics plays a crucial role in advancing our understanding of BC biology, identifying potential biomarkers and therapeutic targets and guiding personalized treatment approaches.

#### 3.1.3. Epigenomics

Epigenetic modifications, which are extrinsic to the genomic sequence itself, are heritable, reversible alterations in histones or DNA that regulate gene activities. Epigenetic dysregulations are widely linked to human diseases, including cancer. Tumor development and progression are significantly influenced by epigenetic changes and the study of epigenomics has been extensively applied to BC research.

- Identification of gene-specific epigenetic alterations

The DNA methylation status of genes related to breast carcinogenesis has been intensively investigated, and a total of 4283 differently methylated genes and 1899 differentially expressed genes were discovered in an examination of 802 BCs. In other studies, extensive hypermethylation was identified in the *TWIST*, *RASSF1A*, *CCND2* and *HIN1* genes in breast carcinoma samples [89,90]. New targets are being uncovered, including the hypermethylation of the *WNT1* promoter in patients with metastatic tumors and *RASGRF1, CPXM1*, *HOXA10* and *DACH1* in TBNC [91,92]. *CDH13* and *GSTP1* hypermethylation are also more common in triple-negative and lymph-node-positive BC patients, respectively [93].

Gene methylation affects the expression of particular genes; for example, in HER2+ breast tumors, epigenetic changes such as the hypermethylation-mediated silencing of negative regulators of the WNT pathway have been discovered [94]. Moreover, the most common cause of *BRCA1* silencing in sporadic BC is promoter hypermethylation, particularly in the triple-negative subtype [95].

- Identification of potential biomarkers and therapeutic targets

Today, there is a growing demand for biomarkers and potential therapeutic targets, and the field of epigenetics is one that is both emerging quickly and has the potential to be helpful in this regard.

The hypermethylation and hypomethylation of genes are now considered indicators of cancer status, e.g., the hypermethylation of *ALDH1A2*, *ALDH1L1*, *HSPB6*, *MME*, *MRGPRF*, *PENK*, *SPTBN1*, *WDR86* and *CAV2* and *PITX1* hypomethylation are now considered potential biomarkers of EBC [96]. Another study indicated that the methylation status of *RASSF1A*, *CCND2*, *HIN1* and *APC* is a possible BC biomarker, with hypermethylation associated with hormone-receptor-positive phenotypes [97,98]. Today’s tools allow for the discovery of potential gene/miRNA prognostic markers in BC by analyzing freely available transcriptome datasets, such as BreastMark (http://glados.ucd.ie/BreastMark/). Numerous biomarkers have been discovered using this, such as the miRNA hsa-miR-210, which has been connected to a poor prognosis in BC patients [99].

Epigenomic investigation could indeed discover crucial molecular markers associated with BC diagnosis and prognosis. The gene *SPAG6* was recently discovered to have higher promoter methylation in serum samples from women with DCIS and early invasive BC compared to controls with benign illness. *SPAG6* methylation was introduced as a promising blood-borne epigenetic biomarker for minimally invasive BC detection, together with other genes, namely *PER1*, *ITIH5* and *NKX2-6* [100]. Equally, H3K4 acetylation is one of the most critical epigenetic modifications, and its dysregulation has been linked to BC progression, estrogen responsiveness and the epithelial–mesenchymal transition oncogenic pathway [74]. Furthermore, it is a potential predictor of cancer-related pathway deregulation and a therapeutic target for breast cancer management [101].

- Target therapies, treatment response and resistance

Epigenetic studies are therapeutically useful since epigenetic aberrations, unlike genetic abnormalities, are reversible and epigenetic therapy can return them to normal levels.

Cellular senescence, EMT, Hippo signaling, the p53 pathway, AMPK signaling and AMP-activated protein kinase (AMPK) signaling are only a few of the pathways that can be affected by these epigenetic dysregulation mechanisms, ultimately leading to mutations and cancer [102]. Epidrugs are small-molecule inhibitors that work to control abnormal epigenetic modifications by reducing enzymatic activity and targeting specific enzymes [103,104]. Interestingly, over the past few years, several drugs that target epigenetics changes have been developed, such as the DNA methyltransferase NSD3 inhibitors [102], targeted small-molecule inhibitors [105], histone-modifying enzymes and mRNA regulators (such as miRNA mimics and antagomiRs) [106].

BC patients may have alterations linked to therapy responses or resistance, according to an epigenomic study. A differential methylation spectrum was associated with a good response to neoadjuvant chemotherapy, as in the case of hypermethylation of the gene *CDKL2* in TNBC patients or *ESR1* methylation as a good predictor of survival only in patients receiving tamoxifen treatment; it can predict a patient’s receptor status and hormone therapy response, according to Widschwendter et al., and *ARHI* methylation can predict survival in patients not receiving tamoxifen [107,108]. Moreover, low risks of metastasis and mortality are linked to the presence of a breast CpGI methylator phenotype, which is defined by hypermethylation in many genes. Furthermore, *CST6* hypermethylation in cfDNA has been related to prognosis and survival in individuals with operable BC [109].

In general, epigenomics is essential in improving our comprehension of BC biology, locating possible therapeutic targets and directing individualized therapy regimens.

### 3.2. Proteomics

Functional characteristics and post-translational modifications cannot be completely represented by gene expression only [110]. Therefore, proteomics can integrate genomic and transcriptome data [111]. A single pre-mRNA transcript can be spliced into different protein isoforms and modified in various ways after translation to produce multiple distinct proteins from a single gene. These modifications are known as post-translational modifications, or PTMs [112]. Immunohistochemistry, reverse-phase protein arrays, Western blotting and ELISA are a few examples of focused proteomics techniques. Mass spectrometry (MS) imaging, targeted proteomics and next-generation proteomics are examples of MS-based targeted proteomics methods with a wide array of different proteins and PTMs that can be assessed at the same time.

- Characterization of heterogeneity

The advances in the field of proteomics have made possible the analysis of protein abundance, protein–protein interactions, protein function or modification and the heterogeneity of BC [113]. Collectively, 97 BC biosignatures have been reported so far from proteomics studies, including pathways related to ER, p53, CK8/18, Ki-67, PR, cyclin D1, HER-2, CK5/6, cyclin E, BCL2, cyclin E and E-cadherin [81].

The features of the TNBC gene expression profile and proteome were investigated in depth, focusing on the precise mechanism for metastatic growth, adhesion and angiogenesis [114,115]. From these studies, it emerged that phosphorylation is the most prevalent post-translational protein modification, and the specific protein phosphorylation events have a direct impact on the development of TNBC tumors and other BCs; therefore, phosphoproteomics-based MS has been the method of choice in studying protein phosphorylation [112,116]. As an example, Asleh et al. found, in 88 TNBC cases, four proteomic clusters displaying features of basal-immune hot, basal-immune cold, mesenchymal and luminal phenotypes, with different survival outcomes [117]. In this study, not only TNBC but also other subtypes of BC were investigated; meanwhile, another study revealed that 75 Her2-enriched cases could be separated into groups differing in terms of the extracellular matrix, lipid metabolism and immune response features [117].

- Identification of biomarkers and drug targets

With the ability to evaluate quantitatively different proteins, targeted proteomics offers novel methods to verify putative biomarker diagnostic, prognostic or predictive performance in a sizable cohort of clinical samples. MS analysis identified different peptide biomarkers, including fragments of C3, C3adesArg, factor XIIIa, ITIH4, FPA, apoA-IV, fibrinogen, bradykinin and transthyretin, that could be used to define a proteomic landscape for the early diagnosis of BC [118].

Proteomics approaches can also offer more precise diagnoses for known actionable targets, identify new tumor susceptibilities for translation into treatments for aggressive tumors and implicate new mechanisms whereby BC resists treatment. A recent study highlighted that a “proteogenomics” approach for 122 primary breast cancers, when compared to standard BC diagnoses, provided a more detailed analysis of the ERBB2 amplicon, defined better tumor subsets that could benefit from immune checkpoint therapy and allowed a more accurate assessment of the Rb status for the prediction of CDK4/6 inhibitor responsiveness [119].

The use of proteomics has also helped to identify proteins with potential roles in tumor suppression or promotion, such as Maspin and HSP-27, respectively, identified as new therapeutic targets for BC treatment [81].

- Assessment of diagnostic and treatment efficacy

In BC studies, proteomics analysis has been used to identify different breast cancer subtypes and specific protein and expression cases, assess the efficacy of cancer therapies at a cellular and tissular level and even associate distinct proteomic patterns with patient prognosis in BC.

Various proteins have been identified as potential biosignatures connected to the development of BC. By using LC-LTQ/FT-ICRMS (mass spectrometry), a reversed-phase nano-liquid chromatography coupled with a hybrid linear quadrupole ion trap/Fourier transform ion cyclotron resonance mass spectrometer, Semaan et al. identified several phosphoproteins linked to the development of BC, such as specific membrane proteins, cytoplasm or macromolecular complex proteins—including for early diagnosis—retinoic acid receptor alpha and CD14 [112,120]. Another example is the study conducted by Minic and colleagues, which performed phosphoproteomics on three cell line models for BC, MCF10A (normal epithelial cells from mammary gland, non-malignant), MCF7 (estrogen and progesterone-receptor-positive, metastatic) and MDA-MB-231 (TN-negative, highly metastatic). It was found that the ACLY, SIRT1 and SIRT6 enzymes were highly phosphorylated, and hence activated, in BC cells compared to non-malignant cells [121].

Another application of phosphoproteomics is the prediction of the BC response to treatment, as for Paclitaxel, the most used chemotherapy medication. Recently, Mouron et al. conducted a phosphoproteomic screening of 130 HER2-negative female BC cases in treatment with Paclitaxel and found that patients with higher levels of CDK4 and filamin-A phosphorylation had a 90% likelihood of obtaining a pathologic complete response (pCR) to Paclitaxel [122]. Proteins such as TRIM28, HSP90alpha, hnRNP A1, CLTC and myosin-9 were found specifically dephosphorylated in BC cells when treated with Lapatinib, resulting in the slowed development of TNBC [120]. A study based on MS proteomics in a cohort of 113 FFPE samples found that two proteins of the proline biosynthesis pathway, PYCR1 and ALDH18A1, were significantly associated with resistance to treatment, based on pattern dominance. Moreover, the knockdown of PYCR1 reduced the invasion and migration capabilities of BC cell lines and increased the drug sensitivity of orthotopically injected ER-positive tumors in vivo, thus emphasizing the role of PYCR1 in resistance to chemotherapy [123].

Lastly, the use of the isobaric tags for relative and absolute quantification (iTRAQ) labeling-based proteomic approach led to a different expression signature for the three proteins desmoplakin (DP), thrombospondin-1 (TPS1) and tryptophanyl-tRNA synthetase (TrpRS), which were found for relapsed and non-relapsed TBNC tumors. Their overexpression significantly impaired disease-free survival and increased the risk of TNBC patients’ recurrence [124].

Overall, proteomics can provide valuable insights into the biology of breast cancer and help to develop more effective diagnostic and treatment strategies.

### 3.3. Metabolomics

Genomic alterations could also cause changes in metabolic profiles, and these changes eventually could facilitate cancer development [14]. Without ignoring the information offered by genetics, biomarkers can be discovered by examining drug metabolism, including immunometabolism and its connection with the microbiota. Cancer can be considered a metabolic disease brought about by genetic or non-genetic signaling and metabolic abnormalities, with hypoxia, inflammation and changes in metabolism being drivers of carcinogenesis [125]. In fact, since metabolite formation is sensitive to both internal and external stimuli, the metabolome offers the potential to serve as a biological phenotypic probe that can shed light on what occurs in cells [126]. According to recent studies, tumor cells can alter the intracellular metabolism to meet the needs of unchecked proliferation, and the early detection and monitoring of cancer can be performed by detecting aberrant metabolic phenotypes [127]. Metabolomics analysis has been used in BC as an approach to discovering potential pharmacological susceptibility to therapies, such as the antitumor activities of sulforaphane (SFN) in BC patients [77], or to target metabolic limitations [128,129,130].

- Tumor typing and classification

Metabolomics analysis can be used to classify breast cancer based on tumor biology, helping to identify different subtypes and molecular characteristics.

For the classification of BC subtypes, Fun et al. screened 64 differential metabolites between BC and healthy individuals and developed a panel of eight distinct metabolites, including carnitine, lysophosphatidylcholine, proline, alanine, lysophosphatidylcholine, glycochenodeoxycholic acid, valine and 2-octenedioic acid, to classify BC subtypes [131]. The phospholipids of membranes, such as phosphatidylcholines (PC), phosphatidylethanolamines (PEs) and phosphatidylinositols (PIs), as well as sphingomyelins (SM) and ceramides (Cer), were the most increased lipids in tumors, as expected [132]. Triacylglycerols (TGs) were largely unaffected in cancer compared to normal breast tissue, although some were downregulated.

On a collection of 50 tumors, Giskeødegård and colleagues demonstrated that high-resolution magic angle spinning (HR-MAS)–nuclear magnetic resonance (NMR) spectroscopy may be utilized to assess ER and progesterone receptor status, as well as lymph node status, with classifications from 68% to 88% for these three condition markers [133]. Another study found that ER-positive patients had higher alanine, aspartate and glutamate metabolism, decreased glycerolipid catabolism and enhanced purine metabolism when measured using liquid chromatography–mass spectrometry and gas chromatography–mass spectrometry [131].

- Biomarker and therapeutic target discovery

Metabolomics analysis can identify metabolites that serve as potential biomarkers for BC, used for early detection, diagnosis, the monitoring of treatment responses and the assessment of disease progression. According to the type of breast cancer (i.e., HER2+ or ER+), several amino acid transporters, such as SLC1A5, SLC6A14 and SLC7A5, were shown to have different expressions in BC tissue compared to controls [134,135]. Metabolomics analysis found a significant correlation between GPAM expression, patient survival, clinicopathological features, metabolomic and lipidomic profiles and increased levels of phospholipids, particularly phosphatidylcholines [136].

Metabolomics analysis can help to identify metabolic pathways and specific metabolites that are dysregulated in breast cancer, leading to the identification of potential therapeutic targets for drug development and personalized treatment strategies. Brokemöller et al. identified 467 predominant metabolites in BC tissue, using GC-MS-based metabolomics [136]. In total, 57 metabolites showed a significant correlation with high and low glycerol-3-phosphate acyltransferase (GPAM) expression groups, a factor with a key role in lipid biosynthesis and tumor progression. Moreover, N-acetyl-aspartyl-glutamate, an essential tumor-promoting metabolite and a possible target for treatment for high-risk basal-like immune-suppressed (BLIS) subtype BLIS tumors, and S1P, a recognized tumor-promoting intermediate of the ceramide pathway, are two additional potential targets discovered through metabolomics studies in BC [137]. Studies on TNBC also revealed fatty-acid synthase (FASN) as an attractive target for novel tumor-specific therapeutic strategies. Research on its inhibition showed antitumoral effects in both sensitive and chemoresistant cells, which supports the indirect involvement of FASN in TNBC [138]. Moreover, fatty acids (FAs) are downregulated in TNBC relative to other BC subtypes, but, intriguingly, two key enzymes involved in de novo fatty acid synthesis, FASN and acetyl-CoA carboxylase 1 (ACACA), were both found to be upregulated at the protein level in tumors with high quantities of phospholipids containing de-novo-generated fatty acids. Normal cells did not exhibit increased de novo lipid production, making tumor cells an attractive target for novel tumor-specific therapeutic strategies [139,140].

- Insights into metabolic reprogramming

Metabolomics can provide insights into the metabolic reprogramming that occurs in BC cells. It can reveal alterations in metabolic pathways, such as glycolysis, lipid metabolism and amino acid metabolism, which are associated with tumor growth and progression.

The altered energy usage of cancer cells in comparison to normal cells as a result of their elevated rates of proliferation is a defining feature of cancer [141]. Lactate levels are elevated, while glucose levels are lowered in breast cancer cells during glycolysis [142,143,144]. Previously, it was shown that cancer cells only obtained their energy from the metabolism of glucose, and a higher level of lactate was linked to worse 5-year survival rates [145,146]. These results show that the metabolites of carbohydrate metabolism play an important role in the development and spread of BC.

These findings were supported by a second study, which also indicated that BC patients had higher amounts of glycerol, glutamine, glucose-1-phosphate, benzoic acid, palmitic acid, urea, pyrophosphate, serotonin and docosahexaenoic acid than controls. Reduced amounts of 2,3-bisphosphoglyceric acid, fructose, lactamide, N-acetylornithine, lactic acid, maleic acid, cysteine-glycine, glycerol-alpha-phosphate, aspartic acid, pyruvic acid and lactulose were also seen in the BC group [147]. The same observations were supported by the study of Subramani et al., which evaluated also the urea cycle, glutathione metabolism, ammonia recycling, glycine and serine metabolism, phosphatidylethanolamine biosynthesis and arginine and proline metabolism [148].

Yamashita and colleagues performed a metabolomics analysis in 74 BC vs. normal tissues and found differences in the glycolytic pathway and the levels of lactic acid. The authors subclassified BC tissues into hormone receptor-positive and TN cases, and they detected specifically in TNBC the higher expression of ELOVL1 and ELOVL6—involved in the elongation of long-chain fatty acids—and accordingly alterations in the levels of these fatty acids [149].

- Disease monitoring in LB

The metabolomics analysis of biofluids, such as blood, saliva or urine, can provide a non-invasive approach for the monitoring of breast cancer progression and treatment responses. It can detect changes in metabolite profiles that reflect the presence or progression of the disease [150].

For the first time, an ultra-performance liquid chromatography (UPLC)-MS-based technique has been proposed, together with multivariate data analysis, for global saliva metabolomics analysis and BC diagnosis. With hydrophilic interaction chromatography (HILIC) and reversed-phase liquid chromatography (RPLC) separations, 18 possibly useful biomarkers, including glycerol phospholipid compounds, fatty amide, sphingolipid and choline, have been demonstrated to have high accuracy in predicting BC [151]. While acylcarnitine C2 was favorably correlated with disease risk, the amounts of arginine, asparagine and PCs were negatively connected with breast cancer risk in pre-diagnostic plasma samples of 127 metabolites [152].

Serum samples from patients were used in a metabolomics study using NMR spectroscopy to examine the connection between patients’ metabolic characteristics and chemotherapy sensitivity. Several significantly altered metabolic pathways—glycine, serine, threonine, alanine, aspartate and glutamate metabolism and valine, leucine and isoleucine biosynthesis—were identified as potential predictive models to identify three different subtypes of TNBC patients, i.e., those with a pathological complete response (pCR), pathological stable disease (pSD) and pathological partial response (pPR) [153].

This technique has also been used to analyze and compare the serum metabolomes of women with BC before and after 1 year of chemotherapy treatment, revealing significant changes linked to lysine degradation, branched-chain amino acid synthesis, linoleic acid metabolism, tyrosine metabolism and unsaturated fatty acid biosynthesis as the top five altered metabolic pathways [154].

Overall, metabolomics plays a crucial role in breast cancer research by providing insights into the metabolic alterations associated with the disease, identifying potential biomarkers and therapeutic targets and aiding in devising personalized treatment approaches.

### 3.4. Pharmaco-Omics: Pharmacogenomics and Pharmacomicrobiomics

Pharmacogenomics studies how genetic variations affect both pharmacological activity (pharmacodynamics) and pharmacokinetics in individuals, i.e., how a person’s genetic makeup affects his/her reaction to medication [155].

Synthetic lethal pairs or medication combinations to target BC can be identified using genetic mutations, changed transcriptional markers and metabolic alterations [156]. It is reasonable to suppose that some of these alterations will be persistent and accessible for a quantitative evaluation for diagnostic and prognostic purposes, given cancer metabolic reprogramming [157]. Different pathway analysis tools have been developed to compare the effects of gene variants or changes in expression profiles linked to chemotherapy benefits. Herein, we report several examples of how pharmaco-omics has been applied in the BC research field.

- Identification of genetic variants and personalized treatment approaches

Pharmacogenomics analysis can identify genetic variants that affect drug metabolism and responses in BC patients and can guide personalized treatment approaches for BC patients by identifying drugs that are likely to be effective and safe for a particular patient.

A polymorphism in *ABCB1* was indicated as a susceptibility biomarker for Paclitaxel-induced toxicity, but data are still conflicting [158]. Instead, concordant findings point to variants in *CYP2C8* as biomarkers for Taxane-induced neuropathy (TIN) generated by Paclitaxel treatment, or as a biomarker for hematological toxicity brought about by any combination of cytotoxic drugs [158,159].

Mutations located in a hotspot region in the *ESR1* ligand-binding domain include Y537S, Y537N, Y537C and D538G, and these represent more than 80% of the *ESR1* variants associated with acquired resistance to endocrine therapy [160]. These mutations are regarded as the causes of the main resistance mechanism, and they are rare in primary tumors but are reported in more than 20% of cases of recurrence and metastatic cancer in patients treated with endocrine therapy [161]. Since the frequency of the mutations in *ESR1* is higher in metastatic BC than in primary tumors, the assessment of this gene’s variants in plasma cfDNA might help in selecting treatment strategies, e.g., the administration of Fulvestrant in patients with a mutation in *ESR1* was found to improve tumor-free survival [162].

Pharmacogenomics analysis can also help to identify drug targets for BC treatment by revealing the molecular mechanisms underlying tumor development and progression. Indeed, the use of variants in *CBR3* as potential biomarkers for anthracycline-induced toxicity, including cardiac and hematological toxicity, is one of the results of pharmacogenomic BC research [163].

Pharmacomicrobiomics can contribute to the development of personalized medical approaches for breast cancer treatment. The innovative discipline of pharmacomicrobiomics studies the interactions between genetic profiles, drug response variability and toxicity, microbiome variation and drug pharmacodynamics to improve therapeutic efficacy, avoid adverse effects and assess the functional content of commensal bacteria. By considering an individual’s gut microbiota composition and its impact on drug metabolism and treatment responses, tailored treatment plans can be designed to optimize outcomes for patients. The ability to differentiate microbiome profiles when comparing either normal breast tissue vs. BC tissue or the differences in tissues derived from distinct BC subtypes is one of the innovative aspects of microbial analysis in BC [164].

- Influence on drug metabolism and modulation of treatment response or disease recurrence

The gut microbiota can influence the metabolism of drugs, potentially affecting their efficacy and toxicity. Analyzing the microbiota profile alongside other clinical factors can provide valuable information for patient management in terms of treatment and prognosis. Understanding these interactions can help to optimize drug dosing and treatment strategies for BC patients. The gut microbiota has been shown to modulate the efficacy and adverse effects of cancer treatments, including chemotherapy, hormone therapy, targeted therapy, immunotherapy and radiotherapy [165].

Programs such as QIIME 2 (https://qiime2.org/ (accessed on 8 August 2023)) provide a framework for microbiome multi-omics or the concurrent exploration of the microorganisms present in an individual [166]. There is also a link between the breast microbiota and breast carcinogenesis, as well as a relationship between the therapeutic response and drug resistance, which researchers have gradually revealed to exist in both normal breast and BC tissue [167,168]. The responsiveness and toxicity of several cancer treatments may be significantly impacted by the microbiome because it affects host immunity. The innate immune system is taught to recognize microorganisms via pattern recognition receptors (PRRs), which bind to pathogen-associated molecular patterns in bacteria. As a result, certain microbial mechanisms recognized by PRRs in the breast can cause an inflammatory response that inhibits tumor growth and aids in the recruitment of cells that can kill tumors [169].

By studying the interactions between the gut microbiota and different treatments, researchers can gain insights into factors that may influence treatment responses in BC patients. For instance, severe, dose-limiting diarrhea is a common side effect of irinotecan-induced mucositis in up to 30% of BC patients [170]. As a result, microbiome profiling may be used to identify individuals who are at risk of developing mucositis because of irinotecan, and microbiota alterations may result in novel therapeutic options.

Pharmacogenomic analysis can identify existing drugs that are effective for BC treatment by revealing their potential therapeutic targets.

Recently, Oncotype DX [85], a validated and well-known multigene signature test that predicts the risk of recurrence in ER+ lymph-node-negative BC patients who received adjuvant Tamoxifen, and MammaPrint, a validated molecular test that relies on microarrays to evaluate the relative expression of 70 genes primarily involved in cancer regulatory pathways, were developed [171,172]. Overall, pharmacogenomics plays a crucial role in breast cancer research by providing insights into the genetic factors that affect drug metabolism and responses, guiding personalized treatment approaches and identifying potential therapeutic targets for drug development.

### 3.5. Artificial Intelligence (AI) Imaging

AI imaging is a rapidly developing field in breast cancer research and diagnosis. AI-assisted imaging diagnosis provides a more accurate and highly efficient diagnostic model for BC. A new area of medical imaging, radiomics, is based on the extraction and quantification of high-throughput feature data from medical images that cannot be identified by conventional imaging techniques. Radiomics is a non-invasive technique that can be repeated over the follow-up period to infer tumor features [173,174,175]. Although the investigation of genetic expression remains the gold standard, AI imaging creates a link between medical imaging and individualized diagnosis and treatment and has the potential to replace invasive biopsies [176]. Herein, we describe several examples of this technology.

- Improved diagnostic and prognosis accuracy

Radiomics allows us to thoroughly examine tumor heterogeneity, rather than only a tiny portion of the tumor, which is usually the case for genomic and transcriptome profiling [177,178,179]. This idea has been applied to the study of TNBC, considering that the molecular heterogeneity of TNBC subtypes would result in a distinct pattern in MRI images, which can be quantitatively assessed using a radiomics approach, allowing us to diagnose TNBC with high precision [180]. A radiomics approach was used in a retrospective study of 331 cancer cases, where concavity, correlation, roundness and gray mean showed a statistically significant difference. TNBC samples showed smaller concavity, larger roundness and a major gray mean compared to HER2-enhanced and luminal samples [181]. The most statistically significant differences between the molecular subtype “luminal A” and TN tumors were detected using AI imaging [182]. Xiong et al. examined 620 patients with invasive BC, and, in these patients, the radiomics signature effectively predicted disease-free survival (DFS) and outperformed the clinicopathological nomogram [183].

- Identification of biomarkers

AI algorithms can help to identify subtle changes in breast tissue that may be indicative of cancer, improving the diagnostic accuracy and reducing the need for unnecessary biopsies. AI algorithms can also help to identify imaging biomarkers that are associated with BC, such as mammographic breast density. Radiogenomics is a novel emerging omics technique that aims to correlate the phenotype (radio) of lesion imaging with the genotype (genomics), based on the idea that the phenotype is the expression of the genotype [184]. This approach was used to study BC by Woodard et al., who examined the relationships across semantic features—i.e., the Breast Imaging Reporting and Data System (BI-RADS) from mammography and MR imaging—and the clinically accessible genomic assay OncotypeDX’s recurrence risk scores. The authors suggested that the BI-RADS features of mammographic breast density, calcification morphology, mass margins at mammography and MR imaging and non-mass enhancement in MR imaging may be used as imaging biomarkers for the risk of BC recurrence [184].

Another study showed that the MR imaging of breast tumors predicted the underlying expression of genes as detected via RNA-seq; in fact, the researchers found that tumors were smaller and more spherical when immune signaling pathways (T-cell receptor signaling) as well as extracellular signaling pathways (cell adhesion molecules and cytokine–cytokine interactions) were activated [185]. Intratumor heterogeneity in the image enhancement texture was stronger in tumors with higher JAK/STAT and VEGF pathway expression levels. This study of BC highlights the possibility of distinguishing tumors that are more immunologically active [185].

- Improving accuracy of imaging exams

AI algorithms can improve the accuracy of imaging exams by reducing the number of false positives and false negatives, improving the detection of breast cancer [186].

New imaging and analytical techniques must be developed to identify and display the in vivo activity of metabolic pathways. One of the primary instruments used in clinical practice for screening, diagnosis and treatment efficacy assessment is imaging detection, which can show changes in tumor size and texture both before and after therapy. Ultrasound is more adaptable, portable and affordable than a mammogram, but it also depends on the operator’s skill [187]. Magnetic resonance imaging (MRI) is the most expensive method and has low specificity, but it has greater sensitivity than other methods in detecting BC [188,189].

AI algorithms can be used to automate BC screening, reducing the workload of imaging physicians and improving the efficiency of the screening process.

The main challenges in BC screening and imaging diagnosis also include complicated and mutable picture characteristics, varied image quality and inconsistent interpretation by various radiologists and medical institutions. Moreover, the considerable number of images that must be analyzed is challenging for radiologists in terms of time and energy and requires the improvement of computer-aided detection methods and platforms. The development of image-based artificial intelligence (AI)-assisted tumor diagnosis is promising in terms of enhancing the effectiveness and precision of imaging diagnosis. Computer-aided diagnosis (CAD) offers effective automated lesion segmentation, image identification and diagnosis, possibly decreasing radiologists’ labor and increasing the diagnostic precision. The clinical utility of CAD in BC has considerably increased due to advanced image-based artificial intelligence (AI) techniques [190]. AI is able to autonomously recognize, segment and diagnose tumor lesions by building algorithm models from raw image data, demonstrating great application prospects.

Based on the improvements in both screening and diagnostic imaging outcomes, digital breast tomosynthesis (DBT) is emerging as the standard of care for breast imaging. DBT image capture gives higher tomographic detail and improves the section separation or vertical (z-axis) resolution thanks to its wider angular range of X-ray tube motion with access to 1-mm-thick slides [191]. It is noteworthy that DBT detection of invasive carcinoma is associated with a favorable prognosis compared to tubular, papillary and mucinous carcinomas and luminal A molecular subtypes; moreover, DBT has been associated with decreased recall rates compared to digital mammography (DM) [192]. As a result, a decrease in false-positive call back rates of 6 to 67% has been reported with DBT compared to 2D mammography [192,193,194,195,196]. However, an important limitation is the susceptibility to a variety of artefacts, such as 3D reconstruction and slinky or halo artefacts, which increases recall rates and false-positive rates [197].

Overall, AI imaging is a promising tool that can provide valuable insights into breast cancer biology, aid in the development of more effective diagnostic and treatment strategies and improve patient outcomes.

## 4. Conclusions

Innovative omics technologies are continuously in development, thanks to biotechnological advances, allowing researchers to examine multiple data from different sources, e.g., the genome, epigenome, transcriptome, proteome and metabolome, to name a few. Numerous omics technologies, such as bulk and single-cell omics approaches, have made it possible to characterize various molecular layers at previously inaccessible scales and resolutions, offering researchers a comprehensive understanding of the tumor behavior and leading to the possibility of molecularly classifying cancers and enabling a personalized medicine approach.

The application of multi-omics might help to decipher the molecular complexity of BC. Moreover, it can help to classify the different tumor types and BC subtypes and to address survival outcome prediction and BC heterogeneity.

The systematic examination of multiple molecular data at different biological layers is made possible by multi-omics analysis, but it also presents problems in terms of how to obtain useful knowledge from the rapidly growing volume of data. It will be fundamental to develop integrated systems for the management and interpretation of the large amount of multi-omics data, reflecting the various biological fingerprints of the development of BC, particularly of TNBC. In oncology, artificial intelligence has shown the capacity to analyze complementary multi-modal data sets. By using machine learning (ML) approaches on omics profiles, several gene signatures have been identified, but their clinical value is frequently hampered by the restricted interpretability and unstable performance. It is, however, foreseen that this integrated approach will be pivotal in BC management and treatment.

In conclusion, omics technologies, including genomics, proteomics and metabolomics, are valuable tools for breast cancer research. These approaches provide insights into the molecular landscape of breast cancer, allowing for the identification of biomarkers and therapeutic targets (summarized in Table 1) that are pivotal in developing personalized treatment strategies. 

The integrative analysis of multi-omics data will further enhance our understanding of the disease and its underlying mechanisms. Multi-omics will provide a more holistic and comprehensive vision of the breast cancer microenvironment, leading to new insights into the biology of the disease and potential new targets for therapy.

## Figures and Tables

**Table 1:** Summary of principal alterations and biomarkers found by each omics technique.

Omics	Genetic Alterations	Biomarkers
Liquid biopsy		Four polymorphic markers in cfDNA (D13S159, D13S280, D13S282 at region 13q31-33, and D10S1765 at PTEN region 10q23.31) [23].
Transcriptomics	TP53, GAPDH, cyclin D1, HRAS, CDK1, CDC6 and PCNA dysregulated [47]. Activation of ERBB2, FOXM1, *ESR1* and IGFBP2 mechanistic networks [47]. TNBC: expression of ER, PR, GATA3, E-cadherin and multiple cytokeratins [48]. HER2+: high levels of Ki-67, p53, EGFR and the hypoxia marker CAIX [48].	Polyadenylation (APA) influences tumor cell proliferation [52]. ETV6 gene associated with worse prognosis in TNBC [54].
Epigenomics	A total of 4283 differently methylated genes and 1899 differentially expressed genes [89]. Hypermethylation was identified in TWIST, RASSF1A, CCND2 and HIN1 genes [90]. Hypermethylation of the WNT1 promoter in patients with metastatic tumors [91]. RASGRF1, CPXM1, HOXA10 and DACH1 in TBNC [92]. CDH13 and GSTP1 hypermethylation [93].	Hypermethylation of ALDH1A2, ALDH1L1, HSPB6, MME, MRGPRF, PENK, SPTBN1, WDR86 and CAV2 and PITX1 hypomethylation [96]. RASSF1A, CCND2, HIN1 and APC [97,98]. miRNA hsa-miR-210 [99]. SPAG6, PER1, ITIH5 and NKX2-6 [100].
Proteomics	ER, p53, CK8/18, Ki-67, PR, cyclin D1, HER-2, CK5/6, cyclin E, BCL2, cyclin E and E-cadherin [81].	Fragments of C3, C3adesArg, factor XIIIa, ITIH4, FPA, apoA-IV, fibrinogen, bradykinin and transthyretin [118]. Maspin and HSP-27 [81].
Metabolomics	Carnitine, lysophosphatidylcholine, proline, alanine, lysophosphatidylcholine, glycochenodeoxycholic acid, valine and 2-octenedioic acid [131]. Phosphatidylcholines (PC), phosphatidylethanolamines (PEs) and phosphatidylinositols (PIs); sphingomyelins (SM), ceramides (Cer) and triacylglycerols (TGs) [132].	SLC1A5, SLC6A14 and SLC7A5 [134,135]. GPAM [136]. N-acetyl-aspartyl-glutamate, SIP1 [137]. FASN in TNBC [138]. Fatty acids (FAs) in TNBC [139]. Acetyl-CoA carboxylase 1 (ACACA) [140].
Pharmaco-omics	Polymorphism in ABCB1 [158]. Variants in CYP2C8 [159]. Hotspot region in *ESR1* ligand-binding domain, including Y537S, Y537N, Y537C and D538G [160]. Mutations in *ESR1* [162].	Variants in CBR3 [163].
Artificial imaging		Immune signaling pathways (T-cell receptor signaling) and extracellular signaling pathways (cell adhesion molecules and cytokine–cytokine interactions) activated [185]. Higher JAK/STAT and VEGF pathway expression level.

## Data Availability

Not applicable.
